# The parasympathetic and sensory innervation of the proximal and distal colon in male mice

**DOI:** 10.3389/fnana.2024.1422403

**Published:** 2024-07-09

**Authors:** Lixin Wang, Yvette Taché

**Affiliations:** ^1^CURE/Digestive Diseases Research Center, Department of Medicine, Vatche and Tamar Manoukian Division of Digestive Diseases, David Geffen School of Medicine, UCLA, Los Angeles, CA, United States; ^2^Veterans Affairs Greater Los Angeles Healthcare System, Los Angeles, CA, United States

**Keywords:** cholera toxin subunit B, dorsal motor nucleus of the vagus, dorsal root ganglia, mouse, nodose ganglia, pelvic ganglia, proximal and distal colon

## Abstract

**Introduction:**

The distributions of extrinsic neurons innervating the colon show differences in experimental animals from humans, including the vagal and spinal parasympathetic innervation to the distal colon. The neuroanatomical tracing to the mouse proximal colon has not been studied in details. This study aimed to trace the locations of extrinsic neurons projecting to the mouse proximal colon compared to the distal colon using dual retrograde tracing.

**Methods:**

The parasympathetic and sensory neurons projecting to colon were assessed using Cholera Toxin subunit B conjugated to Alexa-Fluor 488 or 555 injected in the proximal and distal colon of the same mice.

**Results:**

Retrograde tracing from the proximal and distal colon labeled neurons in the dorsal motor nucleus of the vagus (DMV) and the nodose ganglia, while the tracing from the distal colon did not label the parasympathetic neurons in the lumbosacral spinal cord at L6-S1. Neurons in the pelvic ganglia which were cholinergic projected to the distal colon. There were more neurons in the DMV and nodose ganglia projecting to the proximal than distal colon. The right nodose ganglion had a higher number of neurons than the left ganglion innervating the proximal colon. In the dorsal root ganglia (DRG), the highest number of neurons traced from the distal colon were at L6, and those from the proximal colon at T12. DRG neurons projected closely to the cholinergic neurons in the intermediolateral column of L6 spinal cord. Small percentages of neurons with dual projections to both the proximal and distal colon existed in the DMV, nodose ganglia and DRG. We also observed long projecting neurons traced from the caudal distal colon to the transverse and proximal colon, some of which were calbindin immunoreactive, while there were no retrogradely labeled neurons traced from the proximal to distal colon.

**Discussion:**

These data demonstrated that the vagal motor and motor and sensory neurons innervate both the proximal and distal colon in mice, and the autonomic neurons in the intermediate zone of the lumbosacral spinal cord do not project directly to the mouse colon, which differs from that in humans.

## 1 Introduction

The origins of parasympathetic and primary sensory neurons projecting to the colon (large intestine) are different from the other parts of the gastrointestinal tract (GI). In humans, parasympathetic neurons innervating the descending/distal colon are located in the sacral spinal cord and the sensory neurons in the dorsal root ganglia (DRG), and the vagal nerves innervate the GI tract up to the transverse colon (Standring, 2008; Câmara and Griessenauer, 2015; Browning et al., 2017; Thompson et al., 2019). However, reports in experimental species indicate that there is variability in the neuronal origins of vagal and pelvic nerves across the segments of the colon (Gonella et al., 1987). In rats, the injection of tracers into the dorsal motor nucleus of the vagus (DMV) or the nodose ganglia anterogradely labeled the efferent or afferent terminals, respectively, in the GI tract to the descending (distal) colon (Berthoud et al., 1990, 1991; Wang and Powley, 2000). In mice, the nodose ganglionic neurons innervate the distal colon less extensively than the proximal colon as assessed by retrograde labeling from the colon (Osman et al., 2023), while anterograde tracing from the nodose ganglia detected sparse vagal afferents in the proximal and mid colon, and none in the distal colon (Spencer et al., 2024). A recent study in mice located the autonomic neurons in the DMV by retrograde tracing from the distal colon (Wang et al., 2024). The preganglionic parasympathetic neurons in the intermediolateral column (IML) of sacral spinal cord were retrogradely labeled from the distal colon in cats (Dorofeeva et al., 2009), but not in dogs (Fukai and Fukuda, 1985) and guinea pig (Olsson et al., 2006), while there is no report in mice and rats. Moreover, the extrinsic neurons innervating the proximal colon in mice have been little investigated compared to the distal colon (Christianson et al., 2006; Tan et al., 2008; Osman et al., 2023; Wang et al., 2024), except the sympathetic innervation to the mouse proximal and distal colon (Smith-Edwards et al., 2021). This has functional significance since these two segments play differential roles in the regulation of colonic motility, transmucosal fluid exchange, local blood flow and immune status. In addition, the mapping of the mouse colonic innervation has relevance to the understanding of the gut-brain interactions in relation with the microbiota (Cryan et al., 2019) and experimental neuromodulation (Salgado Cesar et al., 2023). Mice are also most commonly used experimental species as their genome and epigenome can be precisely manipulated to generate genetically engineered models of human diseases (Tecott, 2003; Navabpour et al., 2020). Therefore, to assess whether the innervation of mouse colon has the same groups of neurons as reported in humans is of important functional and translational applications.

In the present study, the extrinsic parasympathetic and primary visceral sensory neurons were traced to the vagal and/or spinal nuclei, the nodose ganglia and DRG from the proximal and distal colon in the same mouse. This was achieved using Cholera Toxin subunit B (CTb), one of the sensitive and reliable conventional tracers for central and peripheral neural tracing (Sawchenko and Gerfen, 1985; Morecraft et al., 2014; Luchicchi et al., 2021), conjugated to different colored fluorophores, Alexa Fluor (AF) 488 and 555. To identify whether the colon projecting neurons in the pelvic ganglia were cholinergic or catecholaminergic, immunohistochemistry was processed for the labeling of cholinergic neurons with cholinergic acetyl transferase (ChAT) and catecholaminergic neurons with tyrosine hydroxylase (TH). CTb labeled cells projecting to the proximal or distal colon were counted in the dorsal root ganglia (DRG) to assess the segments and peak location of neurons, and in the left and right nodose ganglia and the DMV. Whether there were dual projections to the proximal and distal colon were also examined. Within the colon, the retrogradely labeled intrinsic neurons were mapped in the myenteric plexus as well as calbindin-positive intrinsic projecting neurons.

## 2 Materials and methods

### 2.1 Animals

Adult male mice C57BL/6 J (Stock number 000664, Jackson Laboratories, Sacramento, CA) were maintained two per cage under standard conditions. Animal care and experimental procedures followed institutional ethic guidelines and conformed to the requirements of federal regulations for animal research conduct. All procedures were approved by the Animal Research Committee at Veterans Affairs Greater Los Angeles Healthcare System (animal protocol #07013–17).

### 2.2 Tracers

CTb-AF 488 and 555 (Cat#: 28841 and 28843, Thermo Fisher Scientific United States, thermofisher.com), diluted at 0.5% in 0.01 M phosphate buffered saline (PBS), were aliquoted and stored in -20°C freezer.

### 2.3 Injection of tracers in the mouse colon

**Figure 1:**
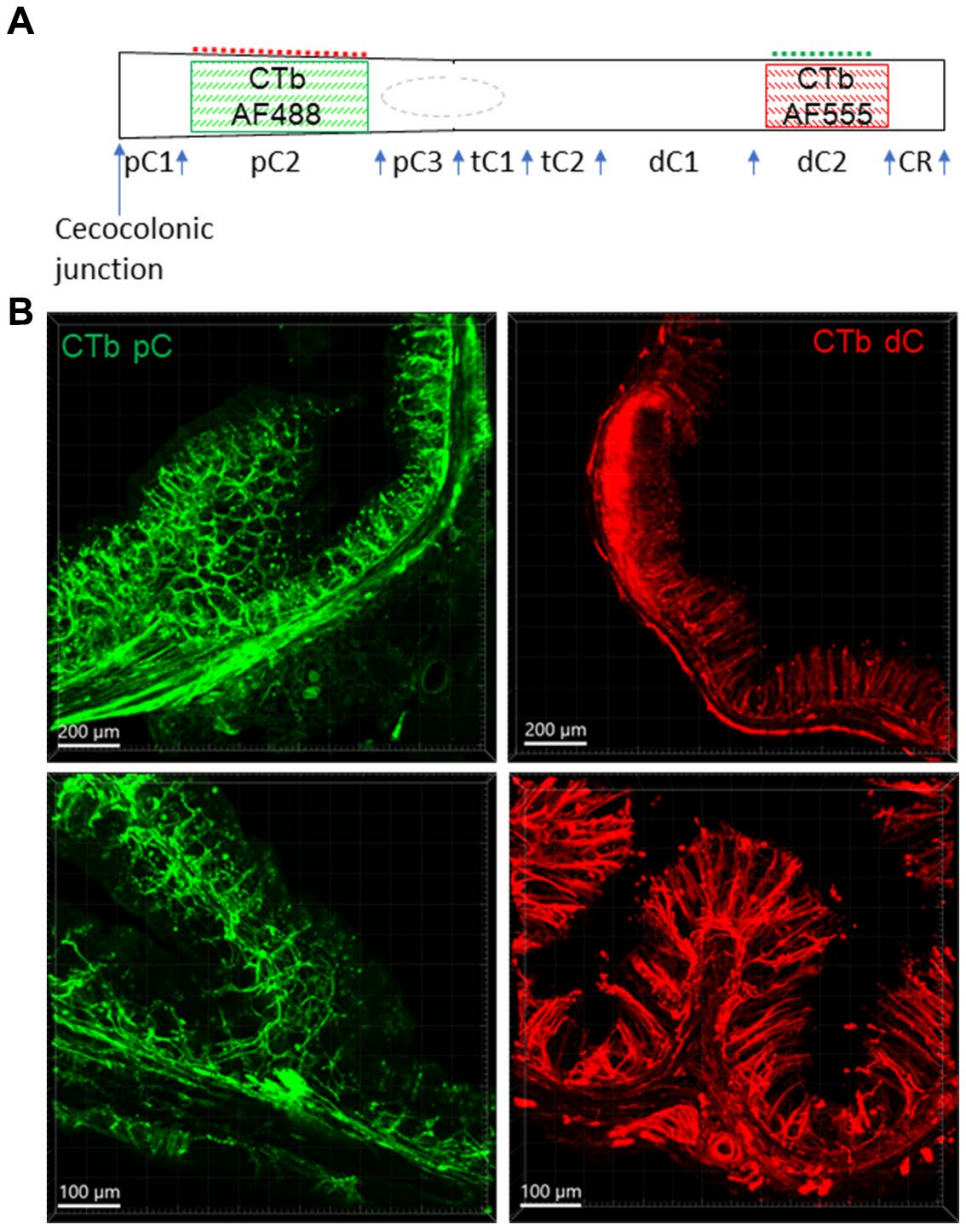
Injection sites of CTb-AF 488 and 555 in the mouse colon. CTb-AF 488 and 555 were injected into the proximal (pC) and distal colon (dC) respectively of the same mice. **(A)** Colored squares illustrate the injection sites in the pC2 and dC2. CTb-AF 488 and 555 were alternatively injected in the proximal and distal colon in different mice. Control injections were made in pC1, pC3 and dC1, as well as mesentery attachments of pC2 and dC2 (dash lines). The rectum is not shown. **(B)** Photomicrographs of sections show the fluorescence of CTb-AF spread to all the layers near the injection sites of the proximal (pC, left) and distal (dC, right) colon at lower (upper panels) and higher magnifications (lower panels). The scales are indicated in the lower corners of each image (same in other figures). tC, transverse colon; CR, colorectum.

Mice (*n* = 28) were anesthetized with 2.5% isoflurane and the abdominal cavity was opened under sterile condition. CTb-AF 488 and 555 were injected in the same mice, respectively in the proximal colon at 0.5–2.5 cm (pC2) from the ileocecal junction, and in the distal colon at 1.5–3 cm (dC2) from the anus (Figure 1A). CTb-AF 488 and 555 were injected alternatively in the proximal or distal colon in different mice. Both sides of each segment of the colonic wall received two injections each of 0.5% CTb solution (1–1.5 μL/injection) as in previous reports (Herrity et al., 2014; Harrington et al., 2019; Wang et al., 2024) using a 35G beveled NanoFil needle (NF35BV-2, World Precision Instrument, Sarasota, FL, United States) (Powley et al., 2014, 2016) connected to a 10 μL Hamilton syringe via a PE-10 tubing. An air bubble separated the tracer from sterile water filling in the tubing and syringe. The injection was performed slowly (1 μL/min), and the needle remained inside the colon wall for 30 s after the completion of the injection to avoid backflow. After the injections, the abdominal wall was sutured in two layers, the muscle layer, and the skin. The analgesic, buprenorphine (0.03 mg/kg; Braeburn, Plymouth Meeting, PA) was injected subcutaneously after the surgery.

Control experiments for injection sites or for possible leakage of tracers into the mesentery are schematically illustrated in Figure 1A. CTb was injected as described above in the rostral and caudal segments of the proximal colon (pC1 and pC3; *n* = 4), rostral segment of distal colon (dC1; *n* = 3), colorectal region (CR, 1 cm from the anus, *n* = 4) and in the mesentery attached to the segments of pC2 (*n* = 3) and dC2 (*n* = 4).

### 2.4 Tissue collection and processing

Mice were euthanized with 5% isoflurane 3–6 days after the tracer injections as previously reported to be optimal time period for maximal intensity (Christianson et al., 2006; Cui et al., 2022), then perfused transcardiacally with 0.9% saline, and the colon was removed. Then, the perfusion was switched to 4% paraformaldehyde in 0.1 M phosphate buffer 2 mL/min for 15 min. The dissected-out colon after saline perfusion was opened along the mesenteric margin, flat-pinned on a Sylgard™ 184 silicone elastomer (Electron Microscopy Science, Hatfield, PA), and fixed in 4% paraformaldehyde in 0.1 M phosphate buffer (PB, pH 7.4) overnight. Additional three mice were perfused with fixative without removal of the colon, and the segments near the injection sites were removed and transversely sectioned at 200 μm as previously described (Wang et al., 2023). Other tissues collected after the fixative perfusion included the medulla oblongata, spinal cord at L6-S1, DRG at T8-S2, and the nodose and pelvic ganglia, were post-fixed overnight. The medulla oblongata and spinal cord at L6-S1 were cryoprotected in 20% sucrose, snap-frozen on dry ice and coronal sections were cut at 50 μm in three sets using a cryostat. One set was mounted on microscopic glass slide, and the other two sets were processed as free-floating sections for immunohistochemistry. Sections of the medulla and spinal cord were mounted onto microscopic slides and sealed by glass coverslip in Vectashield anti-fade mounting medium that has an ideal refractive index, 1.45 (H^-1000^, Vector Laboratories, Burlingame CA). Other samples were mounted inside frames (iSpacer, Sunjin Lab, Taiwan, R.O.C.) on microscopic glass slides (Yuan et al., 2021), and embedded in the Vectashield medium, and then sealed by glass coverslip. The nodose ganglia and DRG as whole ganglia and thick colon sections were mounted in frames with 4 circular wells of 7 mm in diameter and 0.25 mm in depth (IS216, iSpacer). For assessing the injection sites and intrinsic tracing, flat fixed colon with all layers was divided into two halves and sealed on microscopic slides in a frame made from 2 frames of 30 × 22 × 0.5 mm (IS011, iSpacer) cut and pieced together to have a large frame about 45 × 22 × 0.5 mm.

### 2.5 Immunohistochemistry

Sections of the spinal cord L6-S1 were immunostained for ChAT, and the colon for calbindin. The pelvic ganglia were double-immunostained for ChAT and TH. The antibodies information is specified in Table 1. The sections and ganglia were incubated first with primary antibodies in 0.3% Triton-X 100 and 3.5% normal donkey serum in 0.01 M PBS for 2 h at room temperature (RT), and then at 4°C for 2 days. This was followed by the incubation with the secondary antibodies (donkey anti-goat IgG AF 488 or 647 for ChAT and donkey anti-rabbit AF 647 for other primary antibodies) for 2 h at RT. After the immunolabeling, the colonic tissues and spinal cord sections were mounted onto microscope slides, and then sealed in Vectashield anti-fade mounting medium (Vector Laboratories, Burlingame, CA).

**Table 1:** Primary antibodies information.

Primary antibody	RRID^*^	Species	Source	Catalog No.	Dilution
Calbindin	AB_10000340	Rabbit	Swant	CB 38	1:2,000
ChAT	AB_2079751	Goat	Millipore	AB144P	1:500
TH	AB_390204	Rabbit	Millipore	AB152	1:1,000

^*^RRID, Research Resource Identifiers.

### 2.6 Microscopy and cell counting

Microscopic images were acquired in 3D with Z-stacks at 1 μm intervals in Zeiss confocal microscopes (LSM 710 and 880). The image visualization was performed using Imaris 9.8–10.0 for Neuroscientists (Bitplane Inc., Concord, MA). CTb-labeled neurons in the nodose ganglia and DRGs on both left and right sides were automatedly counted in 3D in a whole ganglion using “Spots” module of Imaris. The labeled neurons in the DMV were counted on both sides manually in 6–10 sections of the medulla at the level caudal to the obex between Bregma-8.0 to-7.2 mm (Franklin and Paxinos, 2008). The numbers of labeled neurons are presented bilaterally per section in the DMV.

### 2.7 Statistical analysis

Statistical analysis was performed using SigmaPlot 14 (Systat Software, Inc., San Jose, CA, United States). Data are presented as mean ± SEM. Comparisons between left and right nodose ganglia, and between the proximal and distal colon were performed using Student’s *t*-tests. A *p* value <0.05 was considered significant.

## 3 Results

### 3.1 Injection sites

CTb spread in all layers about 1 cm from the injection sites and showed nerve fibers labeling in the nearby submucosa, enteric plexuses, circular muscular layers, and mucosa (Figure 1B).

### 3.2 Extrinsic retrograde tracing from the proximal and distal colon

#### 3.2.1 Parasympathetic motor neurons

**Figure 2:**
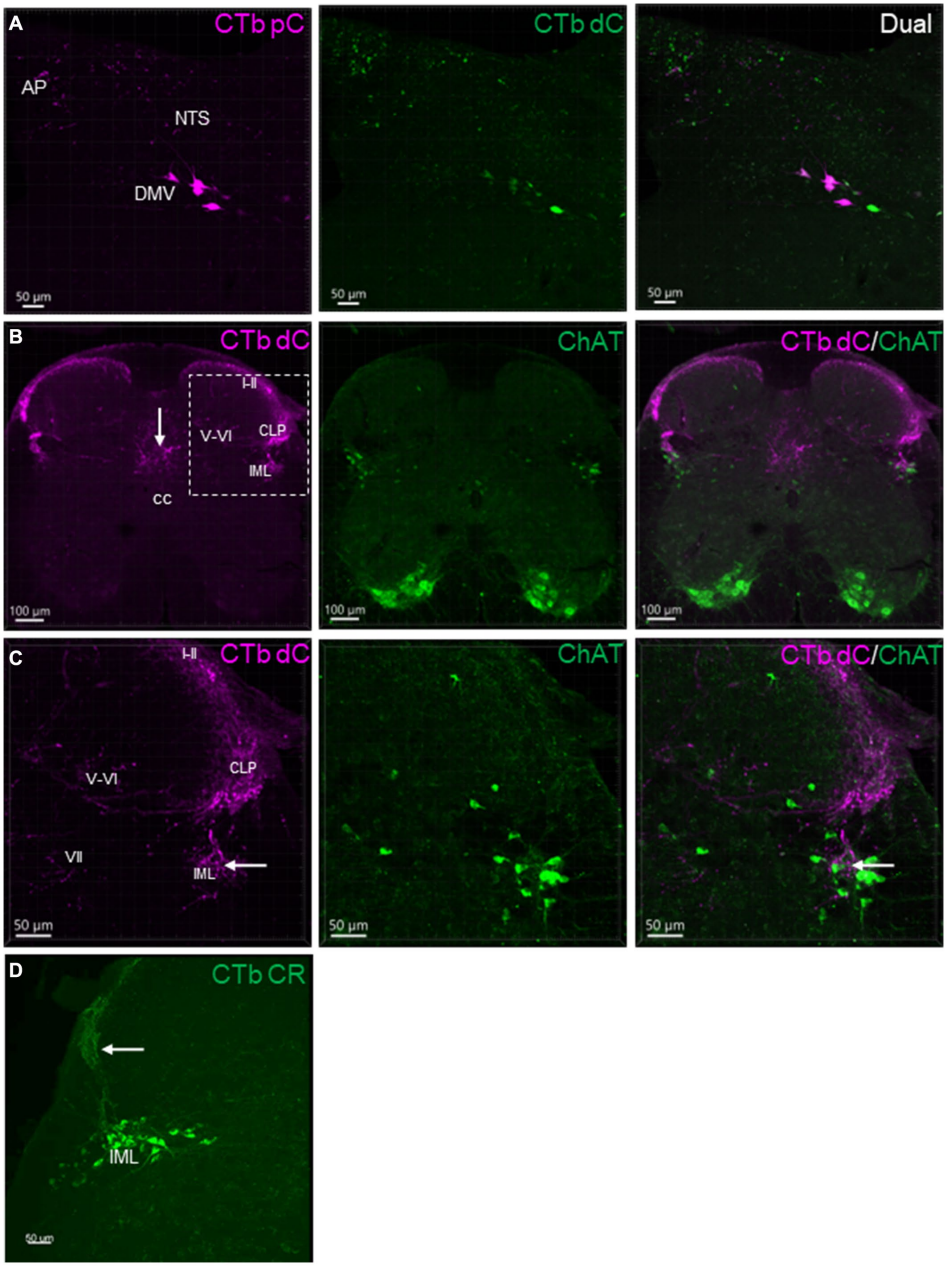
Representative photomicrographs of CTb-AF 488 and 555 labeled neurons and nerve fibers in the sections of dorsal medulla shown **(A)**, and the spinal cord L6 **(B,C)** shown in different channels. CTb-AF 555 and 488 were injected in the proximal (pC) and distal colon (dC) respectively of the same mice. Sections were 50 μm thick. **(A)** Images demonstrate single or dual labeling of CTb. Retrogradely labeled neurons detected in the lateral part of the dorsal motor nucleus of the vagus (DMV), and transganglionic labeled nerve terminals in the nucleus of the tractus solitarii (NTS) and area postrema (AP) from the proximal (magenta, pseudocolor for AF 555) and distal (green) colon. **(B)** Images demonstrate transganglionic labeling of nerve fibers and terminals (magenta) in the spinal cord L6 from the aboral segment of the distal colon, distributed in the laminae I-II and V-VI of dorsal horn, intermediate zone (VII), in the lateral collateral projection (LCP) to the dorsal part of the intermediolateral column (IML) and the dorsal area to the central canal (cc). **(C)** Magnification of the square in B showing the dorsal horn (partial) and intermediate zone. Neurons in the IML were not labeled by CTb injected in the distal colon. The central primary colonic afferents terminated close (arrow) to the cholinergic neurons (ChAT immunoreactive, green) in the IML. **(D)** Neurons in IML labeled by CTB-AF 488 injected in the colorectal (CR) region. Transganglionic labeled nerve fibers in the laminae I-II and CLP (arrow).

**Figure 3:**
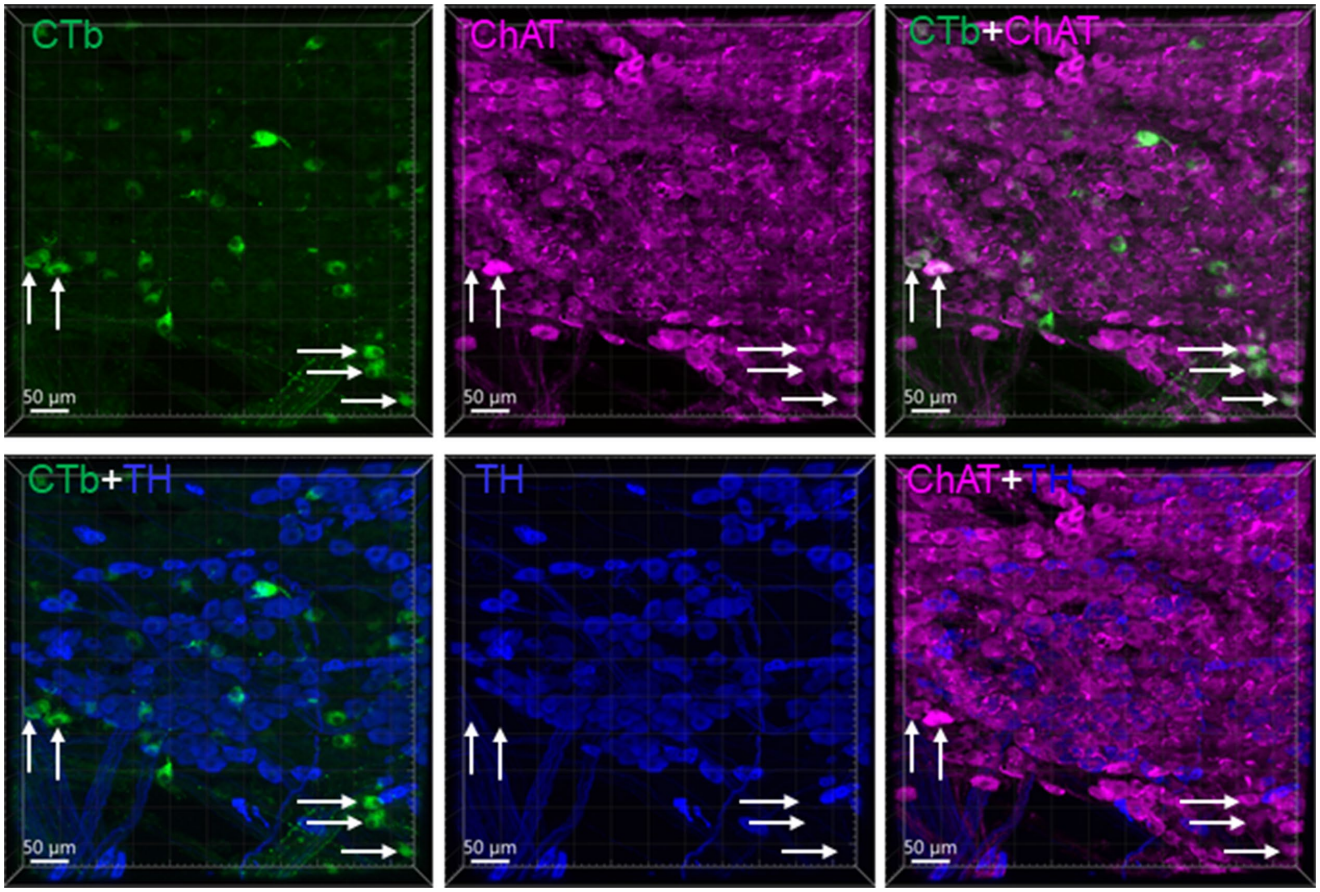
CTb traced neurons in the rostral part of a pelvic ganglion. CTb-AF 488 was injected in the distal colon. The sample with CTb labeling was immunohistochemically stained by choline acetyl transferase (ChAT) and tyrosine hydroxylase (TH) antibodies. Most CTb-labeled neurons (green) were ChAT (magenta) but not TH (blue) immunoreactive. Arrows indicated some of the double cholinergic labeled neurons.

CTb injected into the mouse proximal and distal colon labeled neurons in the DMV of the medulla oblongata (Figure 2). The number of DMV neurons projecting to the proximal colon was significantly higher than that to the distal colon (number/section: 6.0 ± 0.7 vs. 2.2 ± 0.7/section respectively; *n* = 16 mice, *p* < 0.05). Most of them were distributed at the level caudal to the obex between Bregma-8.0 to-7.2 mm in the lateral part of the DMV (Figure 2A). The percentage of neurons double labeled from the proximal and distal colon was 5.9% (0.5 ± 0.2/section). No preganglionic parasympathetic neurons in the IML at L6 and S1 of the spinal cord were labeled by CTb injected in the distal colon (Figure 2B). The pelvic ganglia contained neurons retrogradely labeled by CTb traced from the distal colon, which were located in the rostral enlarged part and middle part of the ganglia. The majority of them were double labeled by ChAT antibody, but not by TH antibody (Figure 3).

#### 3.2.2 Sensory neurons in the nodose ganglia

**Figure 4:**
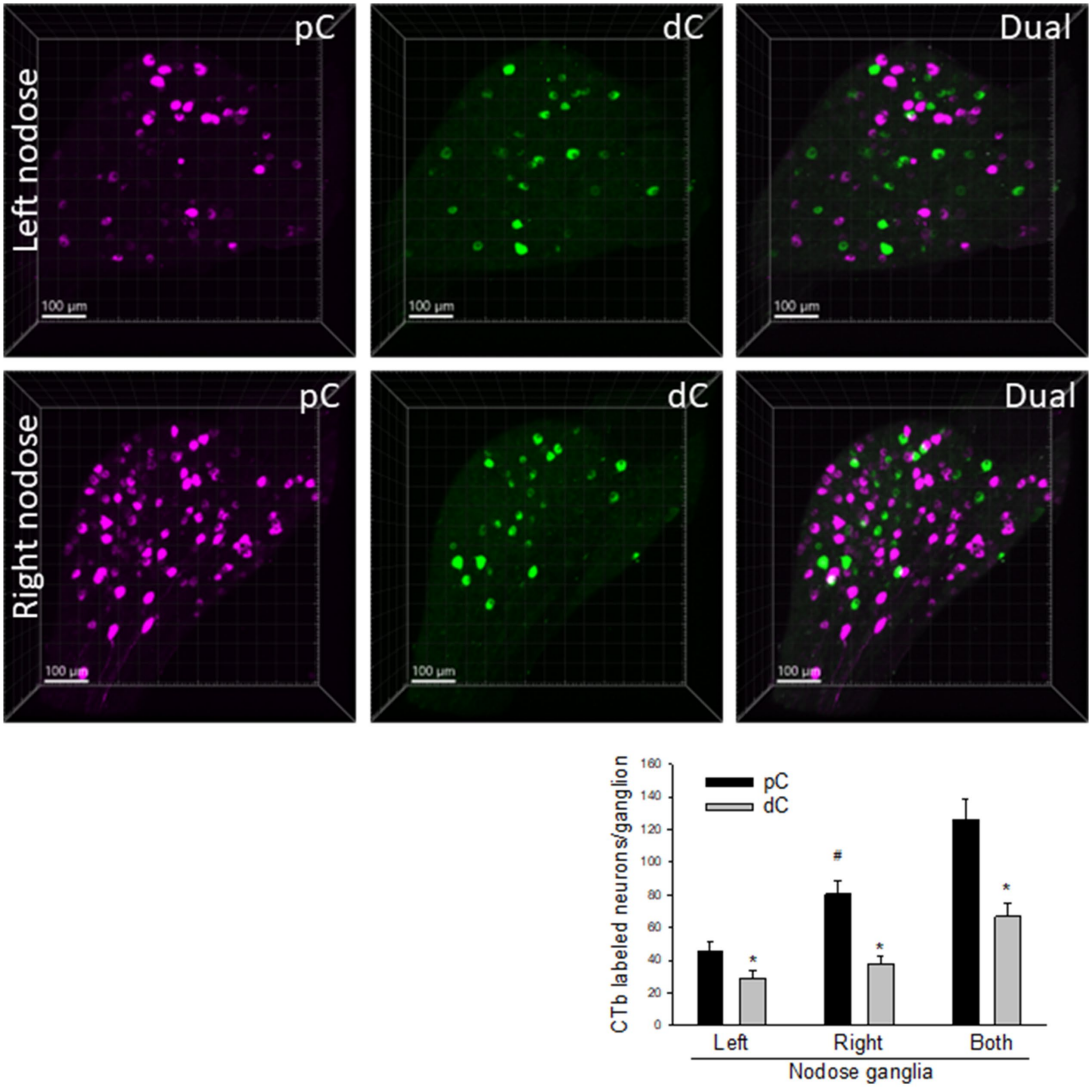
Representative photomicrographs of CTb-AF 488 (green) and 555 (magenta) labeled neurons in the left and right nodose ganglia. CTb-AF 555 and 488 were injected into the proximal (pC) and distal colon (dC) respectively of the same mice. The whole ganglia were taken in 3D images. The graph shows cell count to compare the neurons in the CTb-traced neurons from the proximal and distal colon of the left and right nodose ganglia. Data were means ± SEM of the labeled neurons in a whole nodose ganglion of each mouse (*p* < 0.05: * dC vs. pC, # left vs. right nodose from pC; *n* = 16 mice).

The primary vagal sensory neurons in the nodose ganglia were labeled by CTb injected in both proximal and distal colon (Figure 4). The number of nodose neurons projecting to the proximal colon was significantly higher than those to the distal colon (125.9 ± 12.8 vs. 66.7 ± 8.6 neurons/bilateral ganglia respectively, *n* = 16, *p* < 0.05). The right nodose ganglion contained retrogradely labeled neurons from the proximal colon in a greater number compared to the left nodose (80.4 ± 8.2 vs. 45.6 ± 5.6 neurons/ganglion, *n* = 16, *p* < 0.05), while there was no significant difference compared to those retrogradely labeled from the distal colon (right: 37.6 ± 4.9 vs. left: 29.1 ± 4.2 neurons/ganglion; *n* = 16, *p* > 0.05). The double labeled neurons were 1.9% in the nodose ganglia.

#### 3.2.3 Sensory neurons in the DRG and central terminals

**Figure 5:**
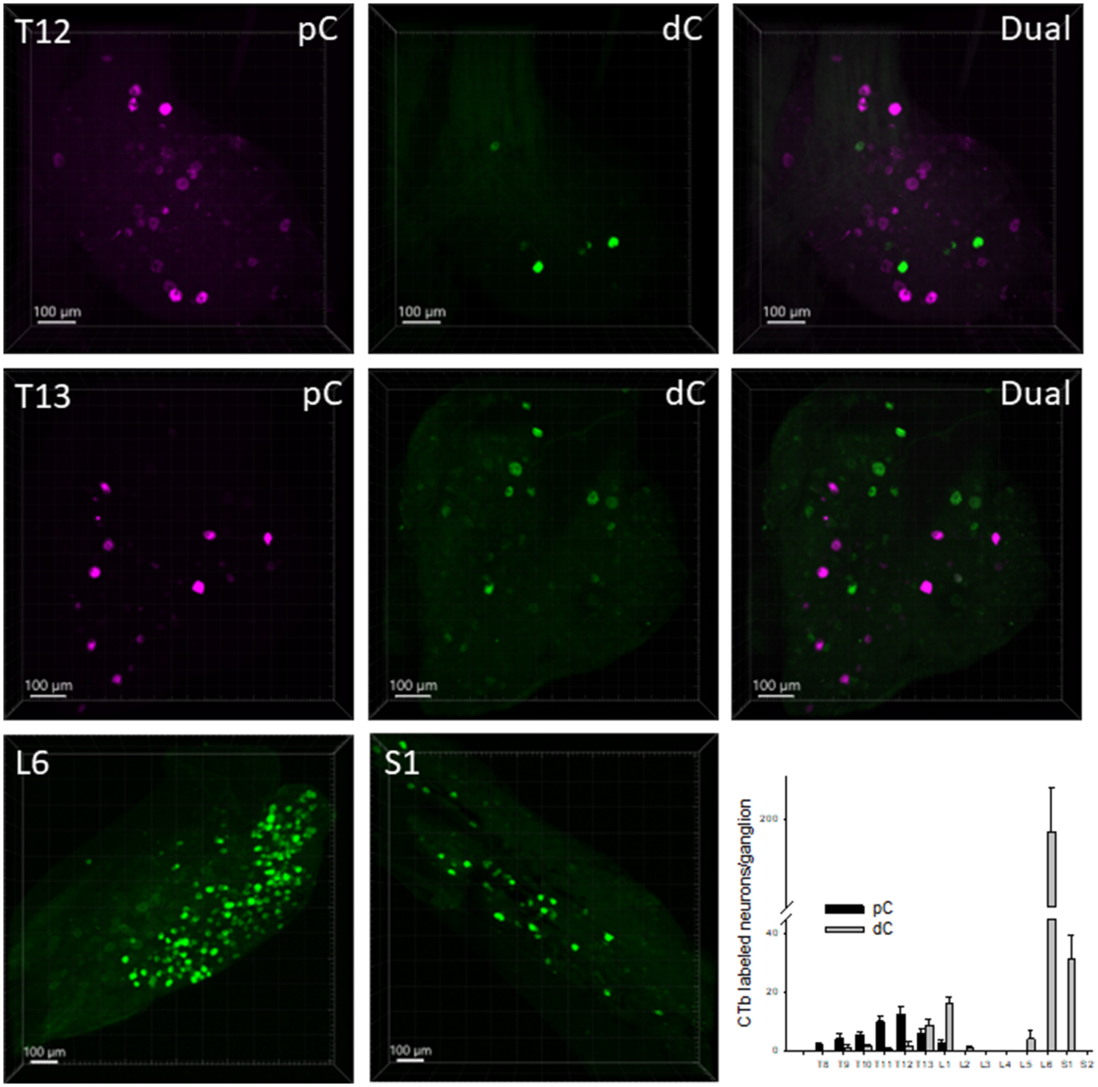
Representative photomicrographs of CTb-AF 555 (magenta) and 488 (green) labeled neurons in the dorsal root ganglia (DRG). CTb-AF 555 and 488 were injected into the proximal (pC) and distal colon (dC) respectively of the same mice. The photomicrographs show in the whole ganglia taken in 3D, the CTb-labeled neurons in the DRG at T12, T13, L6 and S1 traced from the proximal and distal colon. The bar graph illustrates the segmental distribution of labeled neurons. Data are means ± SEM of labeled neurons per ganglion (*n* = 14 mice/DRG segment).

CTb-labeled neurons retrogradely from the proximal colon were located in the DRGs T8-L1 with comparatively more in T11-12 DRGs, a few at T8-T10 and L1 and none at L2-S2 (Figure 5). The main harbors of CTb-labeled primary spinal sensory neurons projecting to the distal colon were in DRG L6 with much less numbers in the DRGs S1 and T13-L1, and a few scattered at T10-12 and L5 (Figure 5). The percentage of dual retrogradely labeled DRG neurons to the proximal and distal colon was 0.8%.

In the spinal cord at L6 level, after CTb injected in the aboral segment of distal colon, transganglionic labeled primary sensory nerves terminated in the lamina I-II of the dorsal horn, intermediate zone, and the area dorsal to the central canal (Figure 2). There were also CTb labeled nerve fibers projecting in the lateral collateral pathway into the dorsal part of IML close to ChAT-containing neurons (Figures 2B,C).

### 3.3 Intrinsic labeling

**Figure 6:**
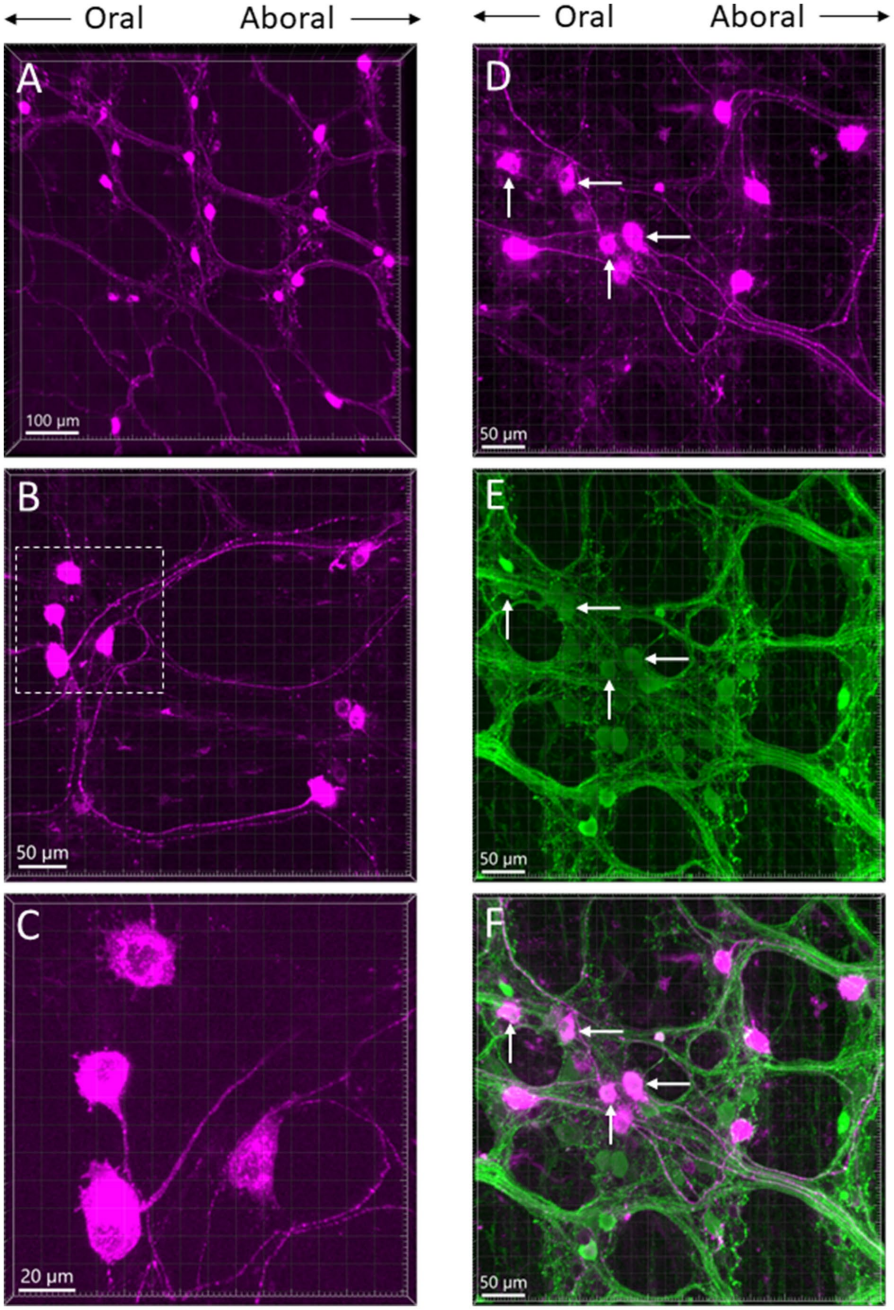
**(A–C)** Photomicrographs of long projection neurons in the transverse colon at different magnifications retrogradely labeled by CTb-AF 555 injected in the aboral segment of the mouse distal colon. All the images are placed in the same oral-aboral directions from the left to right. The neurons with long axons show morphology as Dogiel type II. **(C)** Magnification of the marquee area in panel **B**. **(D–F)** Calbindin immunoreactive neurons and CTb-labeled neurons in the myenteric ganglia of the transverse colon displayed by single labeling of CTb (**D**, magenta), calbindin (**E**, green) and double labeling **(F)**. Arrows indicate some of the dual labeled neurons.

The myenteric and submucosal plexuses were labeled in areas about 1 cm from the needle trail. The long projecting neurons were found in the myenteric plexus rostral to the injection sites when CTb was injected in the distal colon (Figures 6A–D). Retrogradely labeled neurons were traced orally up to the transverse colon and caudal portion of the proximal colon. They were scattered large cell bodies with bright fluorescence and a long axon projecting caudally. Their morphology looked similar to Dogiel type II neurons (Figures 6C,D). Calbindin immunoreactivity was detected in some of the CTb-labeled neurons (Figures 6D–F). When CTb was injected in the proximal colon, there were no long projecting neurons observed in the transvers and distal colon.

### 3.4 Injection controls

When CTb was injected in the mesentery attached to the proximal and distal colon, the distributions of retrograde labeled neurons were in the same locations as the injections in the colon wall of the same segment. Injections in the rostral and caudal segments (as illustrated in Figure 1A) of proximal colon (pC1 and pC3, *n* = 3 mice/group), or rostral half of distal colon (dC1, *n* = 5 mice), also resulted in the same locations of CTb labeled neurons than by injections in pC2 or dC2.

Neurons in the IML were retrogradely labeled when the tracer was injected in the colorectal region (*n* = 4; Figure 2D).

## 4 Discussion

The present study provides insight to the extrinsic parasympathetic motor and primary sensory neurons projecting to the proximal colon that was little investigated in comparison to the distal colon in the same mouse. We showed that the origins of neurons innervating the proximal and distal colon were both from the DMV with a small percentage being dually labelled, while there were no direct projections from the preganglionic parasympathetic neurons in the spinal cord to the mouse colon. The pelvic ganglia contained cholinergic neurons innervating the distal colon. The primary sensory afferents from the proximal colon had neuronal bodies in the nodose ganglia of vagus, and in the thoracolumbar DRGs, while those to the distal colon were mostly located in the lumbosacral and to a smaller extend to thoracolumbar DRGs, as well as in the nodose ganglia. The proximal colon received more vagal motor and sensory projections than the distal colon. The right nodose ganglion harbored a higher number of neurons projecting to the proximal colon than the left nodose ganglion.

### 4.1 Preganglionic parasympathetic neurons

We found that CTb injected in the mouse proximal and distal colon both retrogradely labeled motor neurons in the DMV. A recent report also demonstrated efferent projections from the DMV to the mouse distal colon (Wang et al., 2024). This is consistent with previous reports in rats showing that the vagal nerves project to the distal colon as traced by anterograde labeling from the DMV (Berthoud et al., 1990, 1991; Powley, 2000). Other studies in rats using retrograde tracing from the distal colon also found labeled neurons in the DMV (Altschuler et al., 1993). Collectively the present and previous data indicate that the vagal preganglionic parasympathetic innervation of the distal colon is the same in mice and rats and differs from that in human colon where the vagal nerves innervate the proximal half, but not the descending/distal colon (Standring, 2008; Câmara and Griessenauer, 2015; Browning et al., 2017).

The vagal efferent neurons to the mouse proximal and distal colon were located mainly in the lateral part of caudal DMV, which was similar to previous reports in rats and mice (Altschuler et al., 1993; Kamitakahara et al., 2017). CTb injected into the proximal colon was also traced in the lateral DMV neurons bearing MET tyrosine kinase receptor reporter mice, and these neurons were entirely in the DMV caudal to the obex (Kamitakahara et al., 2017). This localization is related to motor neurons contributing to the celiac branch of the vagus which have been located in the lateral DMV (Fox and Powley, 1985; Norgren and Smith, 1988; Altschuler et al., 1993).

The preganglionic parasympathetic neurons of the lumbosacral spinal cord in mice were not retrogradely labeled when CTb injection was in the distal colon, but appeared when injections were performed in the colorectal region. The recent report also observed in mice that neurons in the sacral parasympathetic nuclei were labeled only by CTb into the colorectal region (Wang et al., 2024). In experimental animals, the direct lumbosacral spinal autonomic projections to the distal colon were reported inconsistently among species, and their existence was shown in cats (Dorofeeva et al., 2009), and rabbits (Bessant and Robertson-Rintoul, 1986), but not in dogs (Fukai and Fukuda, 1985) and guinea pigs (Olsson et al., 2006). After injection of HRP into the rabbit distal colon, labelled cells were observed in sacral spinal cord segments S2-S5, while there were no labelled cells in the DMV (Bessant and Robertson-Rintoul, 1986). The neurons in the IML of the lumbosacral spinal cord were traced projecting to the rectum of guinea pigs (Olsson et al., 2006), and cats (Dorofeeva et al., 2009). We did not do injections in the rectum, however, CTb retrogradely labeled IML neurons when the injection sites were in the colorectal region. In humans, spinal cord S2-S4 segments harbor parasympathetic efferent neurons with axons terminating in the pelvic ganglia, and the myenteric and submucosal plexuses of the descending colon and rectum (Gaskell, 1886; Parent, 1996). Taken together, the relevance of the sacral preganglionic efferent innervation of the distal colon and rectum showed differences between humans and other experimental animals, particularly in rats and mice, most commonly used mammalian animals for biomedical research. These neuroanatomical differences in the pattern of parasympathetic innervation of the distal colon should be considered when assessing vagal and spinal neuromodulation of the colon in humans based on data in rodent models.

### 4.2 Postganglionic autonomic neurons

We did not investigate the locations of postganglionic sympathetic neurons since they were well established in mice (Smith-Edwards et al., 2021), rats (Luckensmeyer and Keast, 1994) and guinea pigs (Olsson et al., 2006). Reports in mice showed that CTb injected in the proximal and distal colon traced the sympathetic neurons in the celiac, inferior mesenteric and pelvic ganglia (Smith-Edwards et al., 2021). In rats, similar distributions were reported in the prevertebral and lower paravertebral ganglia, when retrograde labeling tracers were injected in different colonic segments. Namely, the majority of neurons located in the celiac-superior mesenteric ganglia project to the proximal colon (Luckensmeyer and Keast, 1994; Quinson et al., 2001) while those in the inferior mesenteric ganglia to the distal colon (Luckensmeyer and Keast, 1994).

In the present study, we showed that the pelvic ganglia contained CTb labeled neurons traced from the mouse distal colon. Similarly, the rat distal colon received moderate supply from the pelvic ganglia as investigated using retrograde tracing of Fast Blue (Luckensmeyer and Keast, 1994), or anterograde tracing of DiI (Luckensmeyer and Keast, 1998). We found that the CTb-labeled neurons projecting to the mouse distal colon were cholinergic, but we did not detect retrogradely traced catecholaminergic neurons. The pelvic ganglia were considered to include both sympathetic and parasympathetic neurons, as the thoracolumbar sympathetic nerve fibers, hypogastric and pelvic nerves terminate in the pelvic ganglia in humans (Kuntz and Moseley, 1936) and in rats (Keast, 1995), and the pelvic ganglia bear both TH-and ChAT-immunoreactive neurons in mice (Wanigasekara et al., 2003), rats (Keast, 1995) and guinea pigs (Olsson et al., 2006). DiI-labeled neurons innervating the distal colon were TH-positive in guinea pigs (Olsson et al., 2006). The mixed ChAT-and TH-immunoreactive neurons indicate possible integration of the pelvic organs functions (Luckensmeyer and Keast, 1998). The existence in the pelvic ganglia of distinct lumbar and sacral segmental inputs distinguishes the ganglia from other autonomic ganglia in the cranial sides, which reflects the complexity of the autonomic regulation in the pelvic organs (Keast, 1999; Janig et al., 2017).

### 4.3 Primary colonic sensory neurons

CTb injected in the proximal and distal colon of mice labeled neurons in the nodose ganglia. The data showed significantly more neurons projecting to the proximal colon than to the distal colon, which is consistent with recent studies (Osman et al., 2023; Wang et al., 2024). We also found a significantly higher number of neurons in the right than left nodose ganglion labeled by CTb injected in the proximal, but not after injections in the distal colon. These data support previous findings reported in rats. DiI injected in the nodose ganglia, labelled more afferent fibers from the right than left ganglion terminating in the proximal and transverse colon of rats (the distal colon was not assessed) (Berthoud et al., 1997). Likewise, there was no difference in the numbers of labeled neurons between the left and right nodose while DiI was injected in the rat distal colon (Herrity et al., 2014). The sensory nerves in the proximal colon travel along the mesenteric vessels are derived primarily from the celiac branch of the right vagus and have their neuronal bodies in the right nodose ganglia. A recent study indicates that in mice, the vagal afferents were sparsely observed in the proximal colon and to a lesser extent in the mid colon (10 out of 24 mice) but were absent in the distal colon traced by dextran biotin injected into the nodose ganglia (Spencer et al., 2024). These data contrast with the retrograde tracing from the mouse distal colon in the present and previous studies (Osman et al., 2023). This could partially result from different tracing methodological approaches and protocols.

In mice, the spinal afferent neurons retrogradely labeled from the proximal colon were located in the lower thoracic DRGs, and neurons to the distal colon were numerous in DRG L6, as well as some in S1 and thoracolumbar DRGs. The spinal sensory innervations to the distal colon in experimental animals have been delineated previously by retrograde and anterograde tracings of DRG neurons and showed a similar segmental distributions in mice (Christianson et al., 2006; Tan et al., 2008; Spencer et al., 2014; Wang et al., 2024), rats (Keast and de Groat, 1992; Robinson et al., 2004; Herrity et al., 2014; Kyloh and Spencer, 2014), guinea pigs (Chen et al., 2016) and cats (Keast and de Groat, 1992). Their chemical coding have been established including calcitonin gene-related peptide, substance P and transient receptor potential cation channel subfamily V member 1 (TRPV1) (Keast and de Groat, 1992; Christianson et al., 2006; Tan et al., 2008; Chen et al., 2016). The peripheral axons of neurons in the DRG L6-S1 terminate in all layers of the aboral segment of the mouse distal colon, including the myenteric plexus and blood vessels (Kyloh and Spencer, 2014; Spencer et al., 2014). Previous reports indicated that the central terminals of DRG neurons with peripheral axons to the pelvic organs projected to the sacral spinal cord as shown by transganglionic labeling using horseradish peroxidase conjugated to wheat germ agglutinin (WGA-HRP) applied to the pelvic nerves of rats (Nadelhaft and Booth, 1984) and cats (de Groat et al., 1981), WGA-HRP or CTb-HRP in the urinary bladder and rectum (Pascual et al., 1993; Wang et al., 1998), and CTb in mouse colorectal region (Wang et al., 2024). CTb injected in the mouse distal colon labeled nerve terminals in the same regions, i.e., in the laminae I-II, VII and area X, and nerve fibers from laminae I-II projecting along the lateral margin of the dorsal horn (a.k.a. lateral collateral pathway) into the IML. Both the previous (Wang et al., 2024) and the present studies detected in mice CTb transganglionically traced nerve fibers projecting in the lateral collateral pathway to the ChAT immunoreactive neurons in the lumbosacral IML. Collectively, these data support that in mice, the afferent neurons in the lumbosacral DRG relay signals from the distal colon to the spinal visceral sensory pathways and efferent neurons to the pelvic organs including the rectum and bladder (Brierley et al., 2018), although there is no direct spinal parasympathetic projections to the distal colon.

The DRG primary sensory neurons innervating the mouse proximal colon were less than those to the distal colon, and their segmental distributions were similar as reported on the mouse jejunum (Tan et al., 2008). On average, the T12 DRG contained more neurons, although their numbers were much lower than those in L6 supplying the distal colon. This suggested that the spinal afferents could be less involved than the vagal afferents in the sensory pathways from the mouse proximal colon. It should be noted that a previous study in mice reported different results in the DRG segments using various combinations of Fast Blue (FB), DiI and DiO injected in the mouse proximal and distal colon (Osman et al., 2023). They found a higher percentage of DiI positive neurons in the T8-13 and L5-S2 DRG regions when DiI was injected into the proximal colon compared to the distal colon, although such a difference was not observed using FB (Osman et al., 2023). The discrepancy could result from the use of different tracers (Osman et al., 2023). Taking into consideration the distribution of the spinal nerves, it could be assumed that the lumbosacral DRGs rarely innervate the ascending/proximal colon (Furness et al., 2014), as observed in our study.

The present data in mice also showed that convergent sensory neurons from both proximal and distal colon were rarely observed in the DRG (0.8%) and occurred also at a low percentage (about 2%) in the nodose. This indicates that the sensory pathways of the proximal and distal colon are distinct, providing neuroanatomical support for differential functions (Brierley et al., 2018). The recent report detecting a higher percentage of dual labelled neurons (10–15% in the DRG and 18% in nodose ganglia) innervating the mice colon could be partially related to the lipophilic nature of DiI and DiO used in this study that label axons en passage (Osman et al., 2023).

### 4.4 Intrinsic projecting neurons

CTb injected in the proximal and distal colon labeled neurons in the enteric ganglia. Beside those near the injection spots, long projecting neurons were traced orally up to the caudal portion of the proximal colon from the aboral segment of the distal colon. By contrast, these long projecting neurons towards the proximal colon were not found as CTb did not retrogradely label neurons in the transverse and distal colon when it was injected in the proximal colon. Intrinsic projecting neurons in the colon including cholinergic and nitrergic were established in humans (Porter et al., 2002; Humenick et al., 2021; Yew et al., 2023). In guinea pigs, the DiI-labeled intrinsic projection neurons in the colon were calbindin immunoreactive Dogiel II neurons (Neunlist et al., 2001; Smolilo et al., 2019). This type of neuron was also characterized by electrical recording, intracellular fluorescent dye traced morphologies and immunostained for chemical coding in mice (Furness et al., 2004). In the present mouse study, the morphologies of CTb labeled long projecting neurons were similar to those described before, and calbindin/CTb neurons in myenteric and submucosal ganglia indicate they engage in the complex intrinsic neuronal input from the distal to the proximal colon.

### 4.5 Methodological considerations

The CTb is a non-toxic subunit of cholera toxin, which binds to a cell surface receptor and is internalized, entering axons by active uptake via nerve terminals and transported retrogradely in the axons (Stoeckel et al., 1977; Thiagarajah and Verkman, 2005; Conte et al., 2009a). CTb was used in retrograde tracing from the mouse and rat colon (Christianson et al., 2006; Herrity et al., 2014; Wang et al., 2024) and established to be sensitive (Sawchenko and Gerfen, 1985; Morecraft et al., 2014), reliable and reproducible (Luchicchi et al., 2021). The fluorophore-conjugated CTb enables multi-targets and multi-organ tracing (Conte et al., 2009a,b; Smith-Edwards et al., 2021; Barrett et al., 2022; Kirouac et al., 2022). The retrograde labeling can be combined with immunohistochemical characterization of neurotransmitters in the autonomic (Wang et al., 2024) and sensory neurons (Christianson et al., 2006). However, the labeling efficiency could depend on the quality of the CTb tracers and success of the microinjections, namely successful labeling entails that the tracer in the injected areas reached all layers. Some batches of CTb-AF 488 yielded poor labeling with small numbers of neurons labeled and faint fluorescence. It is also to note that mapping of tracing by tracers injected in the colonic wall could not label all the extrinsic neurons in the colon. The purpose of the study was to demonstrate the origins of the neurons, but not their total amounts. The cell counts of labeled neurons reflect the major source of neurons innervating the colon, not all the colon-projecting neurons.

In our study we can ascertain that the labeling is selective to neurons innervating the colon based on the locations in the DMV, nodose ganglia and DRG segments and differences between the proximal and distal colon. Few studies reported injection leakage of CTb. Lai et al. (2015) did find evidence of leaking when CTb was injected in the sciatic nerves. Due to the continuation of the serosa to the mesentery, the connective tissues under the serosa and the thin wall of the mouse colon, CTb leak might occur to the region where the mesentery is attached to the colon. However, the control experiments performed by injecting CTb into the mesenteric attachment showed the same distributions of labeled neurons. Moreover, CTb injected in the aboral end of proximal colon and oral segment of distal colon showed similar distributions of retrogradely labeled neurons as the injections in the middle proximal colon and caudal distal colon.

## 5 Conclusion

This study presents novel information on the origins of neurons innervating the proximal and distal colon in mice with differences from those reported in humans. First, the vagal motor and sensory neurons project to both the proximal and distal colon, while in humans the vagus has not been found innervating the colonic segments distal to the middle transverse colon. Second, the preganglionic parasympathetic neurons in the intermediolateral column in L6-S1 do not project directly to the mouse distal colon, while in humans, spinal cord S2-S4 contain motor neurons projecting to the caudal half of the colon. The right nodose ganglion bearing more sensory neurons than the left nodose ganglion innervating the proximal colon is consistent with that in rats. The intrinsic long projecting neurons are detected in the transverse and caudal proximal colon towards the distal colon, but not traced from the proximal to distal colon. These findings provide complementary neuroanatomical basis on innervation of the mouse proximal and distal colon, which may have functional implications for vagal neuromodulation in human colon.

## Data availability statement

The original contributions presented in the study are included in the article/supplementary material, further inquiries can be directed to the corresponding author.

## Ethics statement

The animal study was approved by Animal Research Committee at Veterans Affairs Greater Los Angeles Healthcare System (animal protocol #07013-17). The study was conducted in accordance with the local legislation and institutional requirements.

## Author contributions

LW: Conceptualization, Data curation, Formal analysis, Funding acquisition, Investigation, Methodology, Project administration, Supervision, Validation, Visualization, Writing – original draft, Writing – review & editing. YT: Conceptualization, Funding acquisition, Project administration, Supervision, Writing – review & editing.

## References

[ref1] Altschuler S. M. Escardo J. Lynn R. B. Miselis R. R. (1993). The central organization of the vagus nerve innervating the colon of the rat. Gastroenterology 104, 502–509. doi: 10.1016/0016-5085(93)90419-D, PMID: 8425692

[ref2] Barrett M. S. Hegarty D. M. Habecker B. A. Aicher S. A. (2022). Distinct morphology of cardiac-and brown adipose tissue-projecting neurons in the stellate ganglia of mice. Physiol. Rep. 10:e15334. doi: 10.14814/phy2.15334, PMID: 35621038 PMC9136702

[ref3] Berthoud H. R. Carlson N. R. Powley T. L. (1991). Topography of efferent vagal innervation of the rat gastrointestinal tract. Am. J. Phys. 260, R200–R207. doi: 10.1152/ajpregu.1991.260.1.R200, PMID: 1992820

[ref4] Berthoud H. R. Jedrzejewska A. Powley T. L. (1990). Simultaneous labeling of vagal innervation of the gut and afferent projections from the visceral forebrain with dil injected into the dorsal vagal complex in the rat. J. Comp. Neurol. 301, 65–79. doi: 10.1002/cne.903010107, PMID: 1706359

[ref5] Berthoud H. R. Patterson L. M. Neumann F. Neuhuber W. L. (1997). Distribution and structure of vagal afferent intraganglionic laminar endings (IGLEs) in the rat gastrointestinal tract. Anat. Embryol. 195, 183–191. doi: 10.1007/s004290050037 9045988

[ref6] Bessant A. R. Robertson-Rintoul J. (1986). Origin of the parasympathetic preganglionic fibers to the distal colon of the rabbit as demonstrated by the horseradish peroxidase method. Neurosci. Lett. 63, 17–22. doi: 10.1016/0304-3940(86)90005-4, PMID: 3951737

[ref7] Brierley S. M. Hibberd T. J. Spencer N. J. (2018). Spinal afferent innervation of the Colon and Rectum. Front. Cell. Neurosci. 12:467. doi: 10.3389/fncel.2018.00467, PMID: 30564102 PMC6288476

[ref8] Browning K. N. Verheijden S. Boeckxstaens G. E. (2017). The Vagus nerve in appetite regulation, mood, and intestinal inflammation. Gastroenterology 152, 730–744. doi: 10.1053/j.gastro.2016.10.046 27988382 PMC5337130

[ref9] Câmara R. Griessenauer G. J. (2015). “Anatomy of the Vagus Nerve” in Nerves and Nerve Injuries. eds. Shane Tubbs E. R. R. Shoja M. M. Loukas M. Barbaro N. Spinner R. J. (London: Academic Press), 1, 385–397.

[ref10] Chen B. N. Olsson C. Sharrad D. F. Brookes S. J. (2016). Sensory innervation of the guinea pig colon and rectum compared using retrograde tracing and immunohistochemistry. Neurogastroenterol. Motil. 28, 1306–1316. doi: 10.1111/nmo.12825, PMID: 27038370

[ref11] Christianson J. A. Traub R. J. Davis B. M. (2006). Differences in spinal distribution and neurochemical phenotype of colonic afferents in mouse and rat. J. Comp. Neurol. 494, 246–259. doi: 10.1002/cne.20816, PMID: 16320237

[ref12] Conte W. L. Kamishina H. Reep R. L. (2009a). The efficacy of the fluorescent conjugates of cholera toxin subunit B for multiple retrograde tract tracing in the central nervous system. Brain Struct. Funct. 213, 367–373. doi: 10.1007/s00429-009-0212-x, PMID: 19621243

[ref13] Conte W. L. Kamishina H. Reep R. L. (2009b). Multiple neuroanatomical tract-tracing using fluorescent Alexa Fluor conjugates of cholera toxin subunit B in rats. Nat. Protoc. 4, 1157–1166. doi: 10.1038/nprot.2009.93, PMID: 19617887

[ref14] Cryan J. F. O'Riordan K. J. Cowan C. S. M. Sandhu K. V. Bastiaanssen T. F. S. Boehme M.. (2019). The microbiota-gut-brain Axis. Physiol. Rev. 99, 1877–2013. doi: 10.1152/physrev.00018.2018 31460832

[ref15] Cui J. J. Wang J. Xu D. S. Wu S. Guo Y. T. Su Y. X.. (2022). Alexa Fluor 488-conjugated cholera toxin subunit B optimally labels neurons 3-7 days after injection into the rat gastrocnemius muscle. Neural Regen. Res. 17, 2316–2320. doi: 10.4103/1673-5374.337055, PMID: 35259856 PMC9083145

[ref16] de Groat W. C. Nadelhaft I. Milne R. J. Booth A. M. Morgan C. Thor K. (1981). Organization of the sacral parasympathetic reflex pathways to the urinary bladder and large intestine. J. Auton. Nerv. Syst. 3, 135–160. doi: 10.1016/0165-1838(81)90059-X, PMID: 6268684

[ref17] Dorofeeva A. A. Panteleev S. S. Makarov F. N. (2009). Involvement of the sacral parasympathetic nucleus in the innervation of the descending colon and rectum in cats. Neurosci. Behav. Physiol. 39, 207–210. doi: 10.1007/s11055-009-9104-z, PMID: 19142736

[ref18] Fox E. A. Powley T. L. (1985). Longitudinal columnar organization within the dorsal motor nucleus represents separate branches of the abdominal vagus. Brain Res. 341, 269–282. doi: 10.1016/0006-8993(85)91066-2, PMID: 4041795

[ref19] Franklin K. B. J. Paxinos G. (2008). *The mouse brain in Sterostaxic coordinates*. Compact. 3rd Edn. San Diego: Academic Press, Inc.

[ref20] Fukai K. Fukuda H. (1985). Three serial neurones in the innervation of the colon by the sacral parasympathetic nerve of the dog. J. Physiol. 362, 69–78. doi: 10.1113/jphysiol.1985.sp015663, PMID: 4020695 PMC1192882

[ref21] Furness J. B. Callaghan B. P. Rivera L. R. Cho H. J. (2014). The enteric nervous system and gastrointestinal innervation: integrated local and central control. Adv. Exp. Med. Biol. 817, 39–71. doi: 10.1007/978-1-4939-0897-4_3, PMID: 24997029

[ref22] Furness J. B. Robbins H. L. Xiao J. Stebbing M. J. Nurgali K. (2004). Projections and chemistry of Dogiel type II neurons in the mouse colon. Cell Tissue Res. 317, 1–12. doi: 10.1007/s00441-004-0895-5, PMID: 15170562

[ref23] Gaskell W. H. (1886). On the structure, distribution and function of the nerves which innervate the visceral and vascular systems. J. Physiol. 7:1-80.9. doi: 10.1113/jphysiol.1886.sp000207, PMID: 16991419 PMC1484971

[ref24] Gonella J. Bouvier M. Blanquet F. (1987). Extrinsic nervous control of motility of small and large intestines and related sphincters. Physiol. Rev. 67, 902–961. doi: 10.1152/physrev.1987.67.3.902, PMID: 3299412

[ref25] Harrington A. M. Caraballo S. G. Maddern J. E. Grundy L. Castro J. Brierley S. M. (2019). Colonic afferent input and dorsal horn neuron activation differs between the thoracolumbar and lumbosacral spinal cord. Am. J. Physiol. Gastrointest. Liver Physiol. 317, G285–g303. doi: 10.1152/ajpgi.00013.2019, PMID: 31188624

[ref26] Herrity A. N. Rau K. K. Petruska J. C. Stirling D. P. Hubscher C. H. (2014). Identification of bladder and colon afferents in the nodose ganglia of male rats. J. Comp. Neurol. 522, 3667–3682. doi: 10.1002/cne.23629, PMID: 24845615 PMC5853118

[ref27] Humenick A. Chen B. N. Wattchow D. A. Zagorodnyuk V. P. Dinning P. G. Spencer N. J.. (2021). Characterization of putative interneurons in the myenteric plexus of human colon. Neurogastroenterol. Motil. 33:e13964. doi: 10.1111/nmo.13964, PMID: 32839997 PMC7772282

[ref28] Janig W. Keast J. R. McLachlan E. M. Neuhuber W. L. Southard-Smith M. (2017). Renaming all spinal autonomic outflows as sympathetic is a mistake. Auton. Neurosci. 206, 60–62. doi: 10.1016/j.autneu.2017.04.003, PMID: 28566236

[ref29] Kamitakahara A. Wu H. H. Levitt P. (2017). Distinct projection targets define subpopulations of mouse brainstem vagal neurons that express the autism-associated MET receptor tyrosine kinase. J. Comp. Neurol. 525, 3787–3808. doi: 10.1002/cne.24294, PMID: 28758209 PMC5957535

[ref30] Keast J. R. (1995). Visualization and immunohistochemical characterization of sympathetic and parasympathetic neurons in the male rat major pelvic ganglion. Neuroscience 66, 655–662. doi: 10.1016/0306-4522(94)00595-V, PMID: 7644029

[ref31] Keast J. R. (1999). Unusual autonomic ganglia: connections, chemistry, and plasticity of pelvic ganglia. Int. Rev. Cytol. 193, 1–69. doi: 10.1016/s0074-7696(08)61778-7 10494620

[ref32] Keast J. R. de Groat W. C. (1992). Segmental distribution and peptide content of primary afferent neurons innervating the urogenital organs and colon of male rats. J. Comp. Neurol. 319, 615–623. doi: 10.1002/cne.903190411, PMID: 1619047

[ref33] Kirouac G. J. Li S. Li S. (2022). Convergence of monosynaptic inputs from neurons in the brainstem and forebrain on parabrachial neurons that project to the paraventricular nucleus of the thalamus. Brain Struct. Funct. 227, 2409–2437. doi: 10.1007/s00429-022-02534-6, PMID: 35838792 PMC9418111

[ref34] Kuntz A. Moseley R. L. (1936). An experimental analysis of the pelvic autonomic ganglia in the cat. J. Comp. Neurol. 64, 63–75. doi: 10.1002/cne.900640104

[ref35] Kyloh M. Spencer N. J. (2014). A novel anterograde neuronal tracing technique to selectively label spinal afferent nerve endings that encode noxious and innocuous stimuli in visceral organs. Neurogastroenterol. Motil. 26, 440–444. doi: 10.1111/nmo.12266, PMID: 24460783

[ref36] Lai B. Q. Qiu X. C. Zhang K. Zhang R. Y. Jin H. Li G.. (2015). Cholera toxin B subunit shows Transneuronal tracing after injection in an injured sciatic nerve. PLoS One 10:e0144030. doi: 10.1371/journal.pone.0144030, PMID: 26640949 PMC4671609

[ref37] Luchicchi A. Pattij T. Viana J. N. M. de Kloet S. Marchant N. (2021). Tracing goes viral: viruses that introduce expression of fluorescent proteins in chemically-specific neurons. J. Neurosci. Methods 348:109004. doi: 10.1016/j.jneumeth.2020.109004, PMID: 33242528

[ref38] Luckensmeyer G. B. Keast J. R. (1994). Projections from the prevertebral and major pelvic ganglia to the ileum and large intestine of the male rat. J. Auton. Nerv. Syst. 49, 247–259. doi: 10.1016/0165-1838(94)90171-6, PMID: 7806776

[ref39] Luckensmeyer G. B. Keast J. R. (1998). Projections of pelvic autonomic neurons within the lower bowel of the male rat: an anterograde labelling study. Neuroscience 84, 263–280. doi: 10.1016/s0306-4522(97)89502-4, PMID: 9522380

[ref40] Morecraft R. J. Ugoliniy G. Lanciegoz J. L. Wouterlood F. G. Pandyax D. N. (2014). “Classic and contemporary neural tract-tracing techniques” in Diffusion MRI - from quantitative measurement to in vivo neuroanatomy. eds. Johansen-Berg H. Behrens T. E. J. (San Diego, USA: Academic Press), 359–399.

[ref41] Nadelhaft I. Booth A. M. (1984). The location and morphology of preganglionic neurons and the distribution of visceral afferents from the rat pelvic nerve: a horseradish peroxidase study. J. Comp. Neurol. 226, 238–245. doi: 10.1002/cne.902260207, PMID: 6736301

[ref42] Navabpour S. Kwapis J. L. Jarome T. J. (2020). A neuroscientist's guide to transgenic mice and other genetic tools. Neurosci. Biobehav. Rev. 108, 732–748. doi: 10.1016/j.neubiorev.2019.12.013, PMID: 31843544 PMC8049509

[ref43] Neunlist M. Michel K. Aube A. C. Galmiche J. P. Schemann M. (2001). Projections of excitatory and inhibitory motor neurones to the circular and longitudinal muscle of the guinea pig colon. Cell Tissue Res. 305, 325–330. doi: 10.1007/s004410100387, PMID: 11572085

[ref44] Norgren R. Smith G. P. (1988). Central distribution of subdiaphragmatic vagal branches in the rat. J. Comp. Neurol. 273, 207–223. doi: 10.1002/cne.902730206, PMID: 3417902

[ref45] Olsson C. Chen B. N. Jones S. Chataway T. K. Costa M. Brookes S. J. (2006). Comparison of extrinsic efferent innervation of guinea pig distal colon and rectum. J. Comp. Neurol. 496, 787–801. doi: 10.1002/cne.20965, PMID: 16628614

[ref46] Osman S. Tashtush A. Reed D. E. Lomax A. E. (2023). Analysis of the spinal and vagal afferent innervation of the mouse colon using neuronal retrograde tracers. Cell Tissue Res. 392, 659–670. doi: 10.1007/s00441-023-03769-3, PMID: 37004577

[ref47] Parent A. (1996). Carpenter’s human neuroanatomy. 9th Edn. Baltimore: Williams & Wilkins.

[ref48] Pascual J. I. Insausti R. Gonzalo L. M. (1993). Urinary bladder innervation in male rat: termination of primary afferents in the spinal cord as determined by transganglionic transport of WGA-HRP. J. Urol. 150, 500–504. doi: 10.1016/s0022-5347(17)35535-0, PMID: 7686986

[ref49] Porter A. J. Wattchow D. A. Brookes S. J. Costa M. (2002). Cholinergic and nitrergic interneurones in the myenteric plexus of the human colon. Gut 51, 70–75. doi: 10.1136/gut.51.1.70, PMID: 12077095 PMC1773285

[ref50] Powley T. L. (2000). Vagal input to the enteric nervous system. Gut 47 Suppl 4:iv30-iv32. doi: 10.1136/gut.47.suppl_4.iv30, PMID: 11076904 PMC1766799

[ref51] Powley T. L. Hudson C. N. McAdams J. L. Baronowsky E. A. Martin F. N. Mason J. K.. (2014). Organization of vagal afferents in pylorus: mechanoreceptors arrayed for high sensitivity and fine spatial resolution? Auton. Neurosci. 183, 36–48. doi: 10.1016/j.autneu.2014.02.008, PMID: 24656895 PMC4058399

[ref52] Powley T. L. Hudson C. N. McAdams J. L. Baronowsky E. A. Phillips R. J. (2016). Vagal intramuscular arrays: the specialized mechanoreceptor arbors that innervate the smooth muscle layers of the stomach examined in the rat. J. Comp. Neurol. 524:1. doi: 10.1002/cne.23950 PMC4784687 26355387

[ref53] Quinson N. Robbins H. L. Clark M. J. Furness J. B. (2001). Locations and innervation of cell bodies of sympathetic neurons projecting to the gastrointestinal tract in the rat. Arch. Histol. Cytol. 64, 281–294. doi: 10.1679/aohc.64.281, PMID: 11575424

[ref54] Robinson D. R. McNaughton P. A. Evans M. L. Hicks G. A. (2004). Characterization of the primary spinal afferent innervation of the mouse colon using retrograde labelling. Neurogastroenterol. Motil. 16, 113–124. doi: 10.1046/j.1365-2982.2003.00456.x, PMID: 14764211

[ref55] Salgado Cesar H. Brognara F. Ribeiro A. B. Lataro R. M. Castania J. A. Ulloa L.. (2023). Autonomic regulation of inflammation in conscious animals. Neuroimmunomodulation 30, 102–112. doi: 10.1159/000530908, PMID: 37232031

[ref56] Sawchenko P. E. Gerfen C. R. (1985). Plant lectins and bacterial toxins as tools for tracing neuronal connections. Trends Neurosci. 8, 378–384. doi: 10.1016/0166-2236(85)90137-7

[ref57] Smith-Edwards K. M. Edwards B. S. Wright C. M. Schneider S. Meerschaert K. A. Ejoh L. L.. (2021). Sympathetic input to multiple cell types in mouse and human Colon produces region-specific responses. Gastroenterology 160, 1208–1223.e4. doi: 10.1053/j.gastro.2020.09.030, PMID: 32980343 PMC7956113

[ref58] Smolilo D. J. Costa M. Hibberd T. J. Brookes S. J. H. Wattchow D. A. Spencer N. J. (2019). Distribution, projections, and association with calbindin baskets of motor neurons, interneurons, and sensory neurons in guinea-pig distal colon. J. Comp. Neurol. 527, 1140–1158. doi: 10.1002/cne.24594, PMID: 30520048

[ref59] Spencer N. J. Kyloh M. Duffield M. (2014). Identification of different types of spinal afferent nerve endings that encode noxious and innocuous stimuli in the large intestine using a novel anterograde tracing technique. PLoS One 9:e112466. doi: 10.1371/journal.pone.0112466, PMID: 25383884 PMC4226564

[ref61] Spencer N. J. Kyloh M. A. Travis L. Hibberd T. J. (2024). Identification of vagal afferent nerve endings in the mouse colon and their spatial relationship with enterochromaffin cells. Cell Tissue Res. 396, 313–327. doi: 10.1007/s00441-024-03879-6, PMID: 38383905 PMC11144134

[ref62] Standring S. (2008). Gray’s anatomy: The anatomical basis of clinical practice. 40th Edn. London: Churchill Livingstone Elsevier.

[ref63] Stoeckel K. Schwab M. Thoenen H. (1977). Role of gangliosides in the uptake and retrograde axonal transport of cholera and tetanus toxin as compared to nerve growth factor and wheat germ agglutinin. Brain Res. 132, 273–285. doi: 10.1016/0006-8993(77)90421-8, PMID: 70259

[ref64] Tan L. L. Bornstein J. C. Anderson C. R. (2008). Distinct chemical classes of medium-sized transient receptor potential channel vanilloid 1-immunoreactive dorsal root ganglion neurons innervate the adult mouse jejunum and colon. Neuroscience 156, 334–343. doi: 10.1016/j.neuroscience.2008.06.071, PMID: 18706490

[ref65] Tecott L. H. (2003). The genes and brains of mice and men. Am. J. Psychiatry 160, 646–656. doi: 10.1176/appi.ajp.160.4.646 12668350

[ref66] Thiagarajah J. R. Verkman A. S. (2005). New drug targets for cholera therapy. Trends Pharmacol. Sci. 26, 172–175. doi: 10.1016/j.tips.2005.02.003 15808339

[ref67] Thompson N. Mastitskaya S. Holder D. (2019). Avoiding off-target effects in electrical stimulation of the cervical vagus nerve: neuroanatomical tracing techniques to study fascicular anatomy of the vagus nerve. J. Neurosci. Methods 325:108325. doi: 10.1016/j.jneumeth.2019.108325, PMID: 31260728 PMC6698726

[ref68] Wang Q. Caraballo S. G. Rychkov G. McGovern A. E. Mazzone S. B. Brierley S. M.. (2024). Comparative localization of colorectal sensory afferent central projections in the mouse spinal cord dorsal horn and caudal medulla dorsal vagal complex. J. Comp. Neurol. 532:e25546. doi: 10.1002/cne.25546, PMID: 37837642

[ref69] Wang F. B. Powley T. L. (2000). Topographic inventories of vagal afferents in gastrointestinal muscle. J. Comp. Neurol. 421, 302–324. doi: 10.1002/(SICI)1096-9861(20000605)421:3<302::AID-CNE2>3.0.CO;2-N, PMID: 10813789

[ref70] Wang H. F. Shortland P. Park M. J. Grant G. (1998). Retrograde and transganglionic transport of horseradish peroxidase-conjugated cholera toxin B subunit, wheatgerm agglutinin and isolectin B4 from Griffonia simplicifolia I in primary afferent neurons innervating the rat urinary bladder. Neuroscience 87, 275–288. doi: 10.1016/s0306-4522(98)00061-x, PMID: 9722157

[ref71] Wang L. Yuan P. Q. Taché Y. (2023). Vasculature in the mouse colon and spatial relationships with the enteric nervous system, glia, and immune cells. Front. Neuroanat. 17:1130169. doi: 10.3389/fnana.2023.1130169, PMID: 37332321 PMC10272736

[ref72] Wanigasekara Y. Kepper M. E. Keast J. R. (2003). Immunohistochemical characterisation of pelvic autonomic ganglia in male mice. Cell Tissue Res. 311, 175–185. doi: 10.1007/s00441-002-0673-1 12596037

[ref73] Yew W. P. Humenick A. Chen B. N. Wattchow D. A. Costa M. Dinning P. G.. (2023). Electrophysiological and morphological features of myenteric neurons of human colon revealed by intracellular recording and dye fills. Neurogastroenterol. Motil. 35:e14538. doi: 10.1111/nmo.14538, PMID: 36740821

[ref74] Yuan P. Q. Bellier J. P. Li T. Kwaan M. R. Kimura H. Taché Y. (2021). Intrinsic cholinergic innervation in the human sigmoid colon revealed using CLARITY, three-dimensional (3D) imaging, and a novel anti-human peripheral choline acetyltransferase (hpChAT) antiserum. Neurogastroenterol. Motil. 33:e14030. doi: 10.1111/nmo.14030, PMID: 33174295 PMC8126258

